# Design and Evaluation of TIM-3-CD28 Checkpoint Fusion Proteins to Improve Anti-CD19 CAR T-Cell Function

**DOI:** 10.3389/fimmu.2022.845499

**Published:** 2022-04-06

**Authors:** Franziska Blaeschke, Eva Ortner, Dana Stenger, Jasmin Mahdawi, Antonia Apfelbeck, Nicola Habjan, Tanja Weißer, Theresa Kaeuferle, Semjon Willier, Sebastian Kobold, Tobias Feuchtinger

**Affiliations:** ^1^Department of Pediatric Hematology, Oncology and Stem Cell Transplantation, Dr. von Hauner Children’s Hospital, University Hospital, Ludwig Maximilian University of Munich (LMU), Munich, Germany; ^2^German Cancer Consortium (DKTK), Munich, Germany; ^3^National Center for Infection Research (DZIF), Munich, Germany; ^4^Center for Integrated Protein Science Munich (CIPSM) and Division of Clinical Pharmacology, Department of Medicine IV, Klinikum der LMU München, Munich, Germany

**Keywords:** CAR T cells, checkpoint fusion proteins, pediatric leukemia, acute lymphoblastic leukemia (ALL), TIM-3, CD19, CD28

## Abstract

Therapeutic targeting of inhibitory checkpoint molecules in combination with chimeric antigen receptor (CAR) T cells is currently investigated in a variety of clinical studies for treatment of hematologic and solid malignancies. However, the impact of co-inhibitory axes and their therapeutic implication remains understudied for the majority of acute leukemias due to their low immunogenicity/mutational load. The inhibitory exhaustion molecule TIM-3 is an important marker for the interaction of T cells with leukemic cells. Moreover, inhibitory signals from malignant cells could be transformed into stimulatory signals by synthetic fusion molecules with extracellular inhibitory receptors fused to an intracellular stimulatory domain. Here, we designed a variety of different TIM-3-CD28 fusion proteins to turn inhibitory signals derived by TIM-3 engagement into T-cell activation through CD28. In the absence of anti-CD19 CAR, two TIM-3-CD28 fusion receptors with large parts of CD28 showed strongest responses in terms of cytokine secretion and proliferation upon stimulation with anti-CD3 antibodies compared to controls. We then combined these two novel TIM-3-CD28 fusion proteins with first- and second-generation anti-CD19 CAR T cells and found that the fusion receptor can increase proliferation, activation, and cytotoxic capacity of conventional anti-CD19 CAR T cells. These additionally armed CAR T cells showed excellent effector function. In terms of safety considerations, the fusion receptors showed exclusively increased cytokine release, when the CAR target CD19 was present. We conclude that combining checkpoint fusion proteins with anti-CD19 CARs has the potential to increase T-cell proliferation capacity with the intention to overcome inhibitory signals during the response against malignant cells.

## Introduction

Adoptive cell therapy using chimeric antigen receptor-(CAR-)modified T cells has induced high initial response rates in patients with acute lymphoblastic leukemia and B-cell lymphoma (1–3). These encouraging clinical studies led to approval of multiple CD19-targeting CAR products in the last couple of years (4–6). However, not all patients benefit from CAR T-cell treatment, and 40–60% experience relapse in the course of their disease (3, 5). Moreover, treatment of solid tumors with CAR T cells has not been broadly effective to date. Major causes for nonresponse and relapse are insufficient CAR T-cell expansion and loss of CAR T-cell persistence as well as mutation or downregulation of the target antigen (7–9). The ability to escape the attack of the immune system is a very particular characteristic of malignant tumors. In order to do so, tumors can utilize and redirect immune checkpoint axes, which are physiologically used to balance T-cell responses between activation and inhibition in order to allow sufficient control of infections while preventing autoimmunity (10). Immune checkpoint blockade has been used to reactivate and redirect antitumor T cells and is currently investigated as single therapy and in combination with anti-CD19 and other CAR specificities (11).

TIM-3 (T-cell immunoglobulin and mucin domain-containing protein 3) is a type I transmembrane protein that belongs to the TIM family of proteins (12). It is expressed on not only activated T cells but also other immune cell types such as natural killer (NK) cells, myeloid cells, and regulatory T cells (Tregs). Interestingly, unlike other checkpoint receptors such as PD-1 (programmed cell death protein 1), TIM-3 has no known inhibitory signaling motifs in the intracellular domain, but five tyrosines that seem to interact with BAT3 and Fyn (13, 14). Upon binding of TIM-3 to its ligands, the tyrosines get phosphorylated, BAT3 gets released from the complex, and TIM-3 starts to inhibit the T-cell activation. Known ligands of TIM-3 include galectin-9, HMGB1 (high mobility group box protein 1), phosphatidylserine, and CEACAM1 (CEA cell adhesion molecule 1) (15–17). While galectin-9 and HMGB1 are soluble ligands that can be secreted by a variety of different cell types, phosphatidylserine expression is induced on apoptotic cells. The most recently discovered ligand, CEACAM1, is a membrane protein expressed on T cells, but also other immune cells and tumor cells such as melanoma. Increased expression of TIM-3 on T cells has been associated with terminal differentiation and dysfunction (18). In a previous work by our group, we identified TIM-3 expression on bone marrow T cells as a marker of dismal prognosis in pediatric ALL patients and showed that TIM-3 overexpression can inhibit antileukemic T-cell responses mediated by Blinatumomab (19). While TIM-3 blockade is currently investigated in multiple clinical trials, mostly in combination with PD-(L)1 blockade, the exact mechanism of TIM-3 blockade is not known yet as it might interfere with multiple cell types.

The combination of immune checkpoint blockade with genetically modified T cells has shown promising results in early clinical trials. However, as the CAR but not the therapeutic antibody is tumor-specific, checkpoint blockade can lead to systemic side effects (20). In order to specifically block inhibitory checkpoint axes only on tumor-specific T cells, checkpoint fusion proteins were developed. These fusion proteins usually consist of the extracellular domains of the inhibitory molecule (e.g., PD-1) fused to stimulatory intracellular domains (e.g., CD28) to redirect inhibitory signals toward T-cell stimulation. In the last couple of years, PD-1-CD28, TIM-3-CD28, and CD200R-CD28 fusion receptors were described (21–26). Here, we describe a systematic evaluation of TIM-3-CD28 fusion protein designs to specifically overcome inhibitory signals with the potential to increase CAR T-cell functionality and persistence.

## Materials and Methods

### Generation of CAR T Cells

Peripheral blood mononuclear cells (PBMCs) were isolated from whole blood using Biocoll separation solution (Biochrom). Next, T cells were purified using CD4/8 MicroBeads (Miltenyi Biotec) according to the manufacturer’s instructions. T cells were cultured in TexMACS GMP media (Miltenyi Biotec) plus 2.5% human AB serum (kindly provided by Prof. Ramin Lotfi, University Hospital Ulm, Institute for Transfusion Medicine and German Red Cross Blood Services Baden-Wuerttemberg-Hessen, Institute for Clinical Transfusion Medicine and Immunogenetics, Ulm) supplemented with 12.5 ng/ml IL-7 and IL-15 (human, premium grade, Miltenyi Biotec). T cells were activated using T Cell TransAct, human (Miltenyi Biotec) per the manufacturer’s recommendation.

Retroviral particles were generated using producer cells (293Vec-RD114) kindly provided by BioVec Pharma. Supernatant was frozen and stored at -80°C.

T cells were washed and transduced two days after activation. Twenty-four-well plates were coated with 2.5ug RetroNectin Reagent (Takara) followed by a 30-min blocking step (2% Albumin Fraction V, Sigma-Aldrich) and one wash step (1:40 dilution of HEPES 1M (Biochrom) in PBS). The viral supernatant was centrifuged on the coated wells (3,000g, 90 min, 32°C) and discarded afterward. T cells were added and centrifuged 450g for 10 min at 32°C. T cells were washed 48 h after transduction and put back into T-cell media, now containing 6 U/ml IL-2 in addition to IL-7/-15. Cellular composition and T-cell phenotype were analyzed by flow cytometry before cells were frozen on day 12 after transduction. Transduction rates were analyzed by flow cytometric staining of c-myc-FITC (Miltenyi Biotec) and TIM-3-BV421 (Biolegend). For assays, transduction rates were adjusted to the construct with the lowest transduction rate by adding untransduced T cells. Effector cell count refers to the number of CAR/fusion receptor-positive T cells. CD19- and/or CEACAM1-transduced K562 cells were used as target cells unless otherwise stated. Multiple T-cell transductions were not performed; T cells were only transduced once in the process.

### Proliferation Assay

T cells were labeled with a CellTrace Violet (CTV) Cell Proliferation Kit (ThermoFisher Scientific) according to the manufacturer’s instruction. Labeled T cells were co-cultured with target cells at a 1:1 effector-to-target (E:T) ratio for 72 h. Percent proliferating cells and absolute cell counts were analyzed using a MACSQuant Analyzer 10 (Miltenyi Biotec).

### Cytotoxicity Assay

After NK cell depletion using CD56 MicroBeads according to the manufacturer’s instruction (Miltenyi Biotec), T cells were co-cultured with CTV-labeled target cells (ThermoFisher Scientific) at different E:T ratios. The absolute number of remaining target cells was evaluated after 48 h using a MACSQuant Analyzer 10 (Miltenyi Biotec) to calculate the killing capacity of CAR T cells.

### Intracellular Cytokine Stain (ICS)

T cells and target cells were co-cultured for 6 h. Two hours after stimulation, 10 ug/ml Brefeldin A (Sigma-Aldrich) was added. Cells were washed and stained after the indicated time. Intracellular cytokine stain for IFN-γ-PE (BD), TNF-α-PacificBlue (Biolegend), and IL-2-APC (BD) was performed using the FIX & PERM cell Fixation and Permeabilization kit (ThermoFisher Scientific) according to the supplier’s information. Intracellular cytokine stains were analyzed using a MACSQuant Analyzer 10 (Miltenyi Biotec).

### Surface Marker Stain

Activation markers were analyzed by flow cytometry 14 h after starting the co-culture of T cells with target cells at a 1:1 E:T ratio. Anti-CD25-PE, anti-CD69-PE-Vio770, anti-CD137-APC, anti-CD8-APC-Vio770, anti-CD4-VioGreen, and anti-c-myc-FITC (all Miltenyi Biotec) and TIM-3-BV421 (Biolegend) were used. Surface marker stains were analyzed using a MACSQuant Analyzer 10 (Miltenyi Biotec).

### CD3 Coating Assays

CD3 coating assays were performed as previously described (21). Briefly, 96-well plates were coated with CD3 monoclonal antibody (HIT3a, ThermoFisher Scientific). Anti-CD3 of 2ug/ml or 0.25ug/ml anti-CD3 were used for ICS or proliferation assays, respectively. Ligands galectin-9 and HMGB1 were added at 200 ng/well. Fusion receptor-positive T cells (0.1e6) were added per well, and proliferation/cytokine stains were performed after 72 and 6 h, respectively.

### Cell Lines

Cell lines were regularly tested for the absence of contamination/mycoplasma and STR-typed. Cell lines were cultured in RPMI (Biochrom) supplemented with 10% fetal bovine serum (FBS, Sigma-Aldrich), 1% penicillin/streptomycin (Gibco, ThermoFisher Scientific), and 1% L-glutamine (Gibco, ThermoFisher Scientific).

### Correlation Analysis (RNA-seq)

Correlation analysis was done using publicly available RNA-seq datasets and the online platform by H.E. Miller, correlationAnalyzeR, (2021), GitHub repository, https://github.com/Bishop-Laboratory/correlationAnalyzeR.

### Statistics

Statistics were performed using GraphPad Prism. Statistical significance was calculated using t-test or one-way ANOVA as outlined in the figure legends. P values: * <0.05, ** <0.01, *** <0.001, **** <0.0001. Mean plus standard error mean is shown unless stated otherwise.

## Results

### Systematic Design of TIM-3-CD28 Fusion Proteins

**Figure 1:**
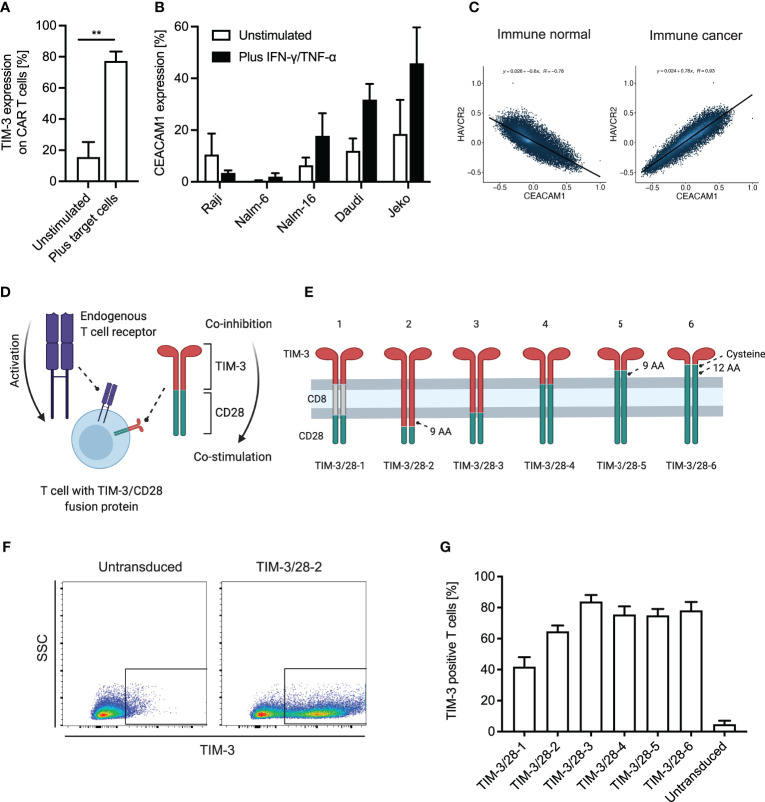
TIM-3 and CEACAM1 expression on T cells/leukemic cells and design of TIM-3-CD28 fusion proteins. **(A)** T cells were retrovirally transduced with an anti-CD19 CAR and co-cultured with CD19^+^ target cells (K562 cells transduced with CD19) for 48 h. TIM-3 expression on CAR T cells was analyzed by flow cytometry. N = 4 individual donors; unpaired t-test was performed. Data are representative of four independent experiments ** < 0.01. **(B)** Leukemia and lymphoma cell lines were either left unstimulated or stimulated with 100 ng/ml IFN-γ and 10 ng/ml TNF-α. CEACAM1 expression was analyzed by flow cytometry. N ≥ 3; unpaired t-test was performed. Data are representative of three independent experiments. **(C)** Correlation of CEACAM1 and TIM-3 (HAVCR2) expression in publicly available datasets was evaluated using the online tool Correlation AnalyzeR (H.E. Miller, correlationAnalyzeR, (2021), GitHub repository, https://github.com/Bishop-Laboratory/correlationAnalyzeR). **(D)** Schematic illustration of a T cell with its endogenous TCR and the TIM-3-CD28 fusion protein that is intended to turn co-inhibition into co-stimulation. **(E)** Schematic illustrations of the six different fusion proteins designed for this study. **(F)** Exemplary flow plot showing transduction of TIM-3/28-2 into primary human T cells as analyzed by TIM-3 expression in flow cytometry. **(G)** Transduction rates as analyzed by flow cytometric staining of TIM-3. N ≥ 3 individual donors. Data are representative of three independent experiments. AA, amino acid; SSC, side scatter. Schematic illustrations created using biorender.com.

We first analyzed TIM-3 expression on anti-CD19 CAR T cells and found a rapid induction of TIM-3 expression already after a single stimulation with CD19^+^ target cells (**Figure 1A**). When analyzing the target cells for the expression of the membrane-bound ligand of TIM-3, CEACAM1, we observed that many leukemia and lymphoma cell lines upregulate CEACAM1 when exposed to Th1 cytokines IFN-γ and TNF-α (**Figure 1B**). As T cells can potentially also express CEACAM1 and contribute to CAR T-cell inhibition through TIM-3, we next checked correlation analyses of publicly available RNA-seq data (**Figure 1C**). We found that in healthy immune cell datasets, TIM-3 (HAVCR2) and CEACAM1 expression levels are inversely correlated hinting toward the fact that TIM-3 and CEACAM1 are usually not expressed simultaneously. In contrast, in immune cancer datasets, TIM-3 and CEACAM1 expressions are strongly correlated. As we did not observe TIM-3 expression on leukemic cell lines (**Supplementary Figure 1A**), we hypothesize that high TIM-3 levels on T cells (or other immune cells) in cancer might correlate with CEACAM1 expression on target or T cells. To transform TIM-3-mediated inhibition into CD28-based co-stimulation (**Figure 1D**), we generated a variety of different TIM-3-CD28 fusion receptors (**Figure 1E**). While fusion receptor 1 (TIM-3/28-1) had a CD8 transmembrane domain, in analogy to CARs, the other fusion receptors 2–6 were comprised of either the TIM-3 or the CD28 transmembrane domain. TIM-3/28-6 had the largest portions of CD28 as it had been shown before that the cysteine in amino acid position 141 of CD28 can increase signaling through the receptor (23). We first retrovirally transduced only the fusion receptors without a CAR into primary human T cells to check expression levels and basic functionality. Transduction rates were analyzed by flow cytometric staining for TIM-3 (**Figure 1F**). While the CD8-containing fusion receptor TIM-3/28-1 showed decreased transduction rates, receptors 2–6 showed robust transduction rates of >60% (**Figure 1G**). When analyzing the geometric mean fluorescence intensity, we observed differences between the TIM-3-CD28 fusion proteins hinting at a different number of molecules per cell based on the type of construct used (**Supplementary Figure 1B**). The transduced T cells consisted mainly of CD8^+^ T cells 12 days after transduction, and the distribution of cell types was not significantly different between the fusion receptors or untransduced T cells (**Supplementary Figure 1C**). The T-cell phenotype was slightly switched toward more effector memory T cells in constructs 2–6 compared to untransduced T cells or T cells transduced with construct 1 (**Supplementary Figure 1D**). Differences in viability throughout the culture were not observed (**Supplementary Figure 1E**). Expansion rates after transduction were comparable (**Supplementary Figure 1F**) with a slight advantage for untransduced T cells.

### TIM-3-CD28-Fusion Proteins With Parts of CD28 for the Hinge Region Exhibit Largest Proliferation Potential and Cytokine Release

**Figure 2:**
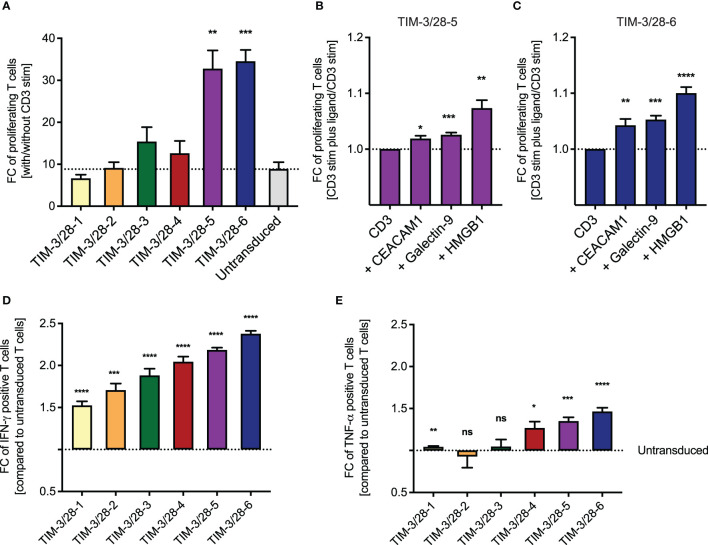
Choosing the best TIM-3-CD28 fusion receptor based on proliferation and cytokine release. **(A)** Fusion receptor-transduced T cells were cultured on anti-CD3-coated plates. Percent proliferating T cells was evaluated by CTV staining and the fold change with/without CD3 stim calculated for each construct. To evaluate the impact of ligand addition, the fold change of proliferating T cells was calculated on CD3 stimulation plus ligand vs minus ligand for TIM-3/28-5 **(B)** and TIM-3/28-6 **(C)**. Fold change of IFN-γ **(D)** and TNF-α **(E)** positive T cells compared to untransduced T cells was analyzed by intracellular cytokine stain for IFN-γ or TNF-α with/without CD3 stimulation. FC, fold change; stim, stimulation. Dotted line represents fold change of untransduced T cells **(A, D, E)** or CD3 stimulation only **(B, C)**. Experiments were performed in two individual donors and technical duplicates. Data are representative of two independent experiments. Unpaired t-test was performed to determine significance. Physiologic expression levels of TIM-3 ligands are shown in **Supplementary Figure 3**. * <0.05, ** <0.01, *** <0.001, **** <0.0001, ns, not significant.

We next tested the different fusion receptor T cells in response to CD3 stimulus and observed that the fusion proteins TIM-3-CD28-5 and TIM-3-CD28-6 showed the highest fold change of proliferating cells (with vs. without CD3 stimulation) as analyzed by CTV staining (**Figure 2A**). Background proliferation without CD3 stimulation was below 20% at that timepoint for all constructs (**Supplementary Figure 2A**). This effect was amplified in the presence of the mostly membrane-bound ligand CEACAM1 and the soluble ligands galectin-9 and HMGB1 (**Figures 2B, C**). When analyzing the cells by intracellular cytokine staining of IFN-γ and TNF-α, we found that all fusion proteins can enhance IFN-γ release while only some of them show an effect in TNF-α secretion (**Figures 2D, E**). TIM-3-CD28-5 and -6 were identified as fusion receptors with highest levels of cytokine release. Background cytokine secretion without CD3 stimulation was below 10% for all constructs (**Supplementary Figures 2B, C**). Cytokine release could only be amplified by the addition of HMGB1 to the culture; the other ligands did not lead to significant changes in cytokine levels tested on the two best-performing constructs (**Supplementary Figures 2D–G**). For reference, physiologic expression levels of TIM-3 ligands were extracted from publicly available RNA-seq datasets and are shown in **Supplementary Figure 3**.

### Generation of Anti-CD19 CAR T Cells With TIM-3-CD28 Fusion Proteins

**Figure 3:**
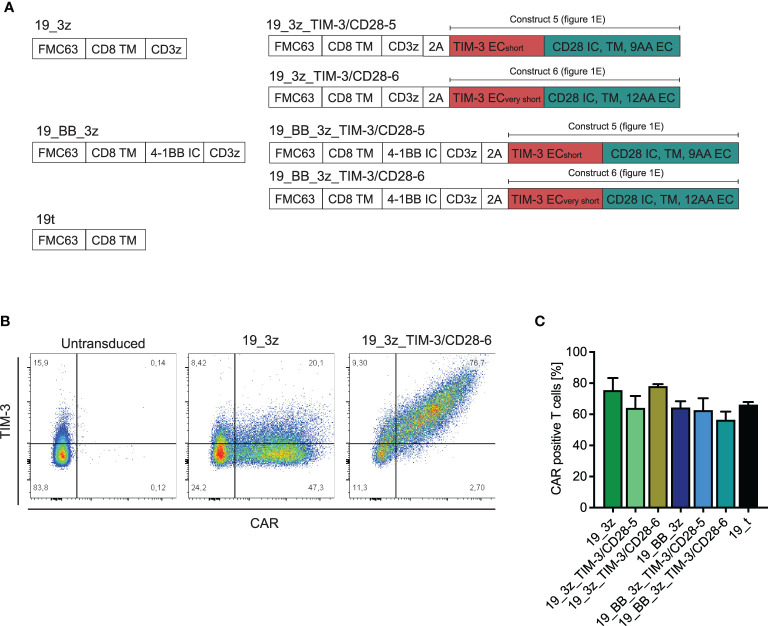
Transduction of primary human T cells with anti-CD19 CARs in addition to TIM-3-CD28 fusion receptors. **(A)** Schematic illustration of transduced CAR T-cell constructs with/without fusion receptors and control 19t. **(B)** Exemplary flow plot showing CAR (myc)/TIM-3 stain in 19_3z CARs with and without fusion protein. **(C)** Transduction rates of CARs with/without fusion proteins as determined by flow cytometric stain for myc. N = 2 individual donors. Data are representative of two independent experiments.

As TIM-3-CD28-5 and -6 were the fusion receptor designs with the highest proliferation and cytokine release, we tested these two constructs in combination with the first- and second-generation CAR T cells. Thus, we created multicistronic constructs with an FMC63-based anti-CD19 CAR and the fusion receptor separated by a 2A cleavage site as depicted in **Figure 3A**. We included a myc tag into the CAR construct for better detection in flow cytometry. Primary human T cells were transduced with the six different CAR constructs plus a truncated anti-CD19 CAR (19t) control, which is lacking intracellular signaling domains. Coexpression of the fusion receptor and CAR was confirmed by flow cytometry (**Figure 3B**). All fusion receptor–CAR combinations showed comparable transduction rates of around 60% (**Figure 3C**) and no difference in viability throughout the culturing process (**Supplementary Figures 4A, B**).

### TIM-3-CD28 Fusion Proteins Can Increase CAR T-Cell Functionality

**Figure 4:**
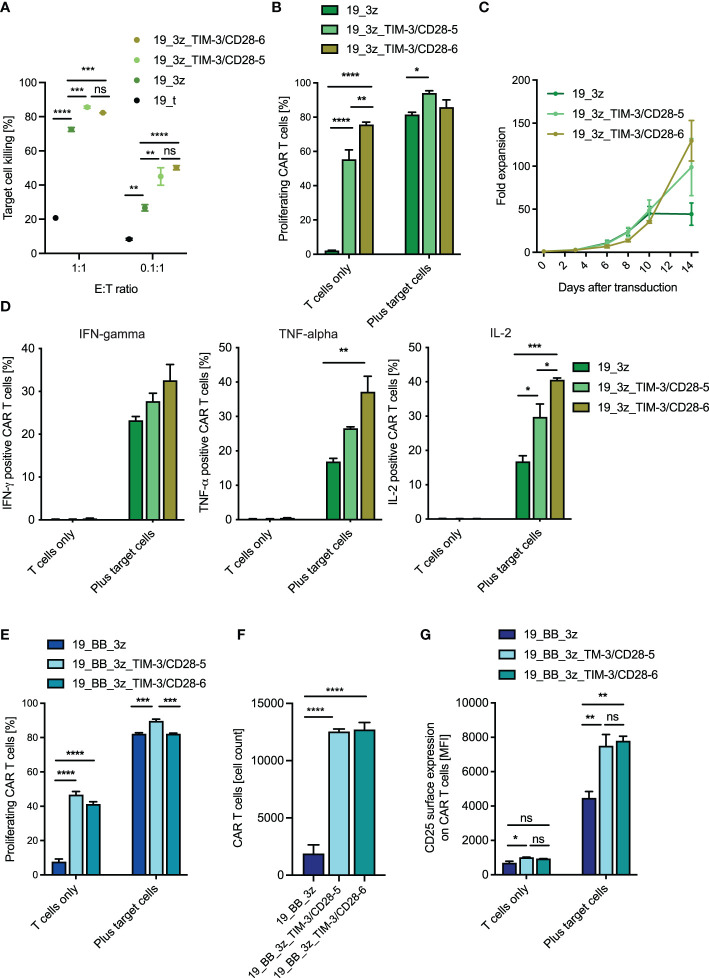
Functionality of anti-CD19 CAR T cells with TIM-3-CD28 fusion proteins. **(A)** Killing of CD19^+^/CEACAM^+^ K562 target cells by first-generation anti-CD19 CAR T cells with/without fusion proteins was calculated after 48 h of co-culture. N = 1 individual donor in technical duplicates for 1:1 E:T ratio and n = 2 individual donors in technical duplicates for 0.1:1 E:T ratio. One-way ANOVA was performed to determine statistical significance. **(B)** First-generation anti-CD19 CAR T cells were co-cultured with target cells (CD19^+^/CEACAM^+^ K562) for 72 h, and percent proliferating cells were analyzed by flow cytometry (CTV). N = 2 individual donors in technical duplicates. One-way ANOVA was performed to determine statistical significance. **(C)** Fold expansion of different CAR T-cell constructs throughout the culture process. Cell count was normalized on the day of transduction. N = 2 individual donors. **(D)** First-generation anti-CD19 CAR T cells with/without fusion proteins were co-cultured with target cells (CD19^+^/CEACAM^+^ K562), and cytokine production was analyzed by intracellular cytokine stain for IFN-γ, TNF-α, and IL-2 6 h after the start of the co-culture. **(E)** Second-generation CAR T cells with/without fusion protein were co-cultured with target cells (CD19^+^/CEACAM^+^ K562), and proliferative potential both in terms of percent proliferating cells **(E)** and absolute CAR cell count **(F)** were analyzed after 72 h. N = 2 individual donors in technical duplicates. One-way ANOVA was performed to determine statistical significance. **(G)** CD25 surface expression was evaluated by flow cytometry 14 h after target cell contact (CD19^+^/CEACAM^+^ K562). N = 2 individual donors, each in technical duplicates. One-way ANOVA was performed to determine statistical significance. Data are representative of two independent experiments **(B–G)**. E:T ratio, effector-to-target ratio; IFN-γ, interferon gamma; TNF-α, tumor necrosis factor alpha; IL-2, interleukin-2. * <0.05, ** <0.01, *** <0.001, **** <0.0001, ns, not significant.

First, we tested whether the fusion proteins can increase the functionality of first-generation CAR T cells as a model system for a suboptimal (and thus optimizable) CAR setting. As expected, we observed a slight decrease in killing capacity of conventional first-generation CARs without fusion protein when the cells got co-cultured with CD19^+^/CEACAM1^+^ compared to CD19+/CEACAM1- target cells (**Supplementary Figure 5A**). Next, we co-cultured first-generation CAR T cells with or without the fusion receptor with CD19^+^/CEACAM1^+^ target cells. Indeed, the addition of the fusion receptor significantly increased the killing capacity of conventional CAR T cells (**Figure 4A**). The same trends were observed after co-culture with CD19^+^/CEACAM^-^ target cells (**Supplementary Figure 5B**). When analyzing the proliferative capacity of the fusion receptor first-generation CAR T cells, we interestingly observed an increased frequency of proliferating fusion protein CARs compared to conventional CARs without the addition of target cells (**Figure 4B**). The same trend was observed when analyzing absolute CAR T-cell counts (**Supplementary Figure 5C**). Looking back at the behavior of the cells during the culturing process, we confirmed this finding as the fusion receptors showed higher proliferative capacity in the absence of target cells (**Figure 4C**). As this result might raise concerns of limited target specificity and potential off-target side effects, we next measured the cytokine release of the fusion protein CAR T cells both in the absence and presence of target cells (**Figure 4D**). Reassuringly, we did not see cytokine release of the fusion protein CAR T cells in the absence of targets, while in the presence of targets, they were able to increase the cytokine release beyond levels detected by conventional first-generation CARs. Moreover, CAR T cells with TIM-3-CD28 fusion protein showed higher levels of CD25 compared to conventional CARs (**Supplementary Figure 5D**), potentially making them an interesting modification in situations of IL-2 competition seen in the presence of regulatory T cells. Surprisingly, we observed higher levels of early differentiation marker CD62L on fusion protein CAR T cells (**Supplementary Figure 5E**), which translated to decreased percentages of terminally differentiated effector cells and higher percentages of early differentiated stem cell-like memory (Tscm) and central memory (Tcm) cells in fusion protein CARs (**Supplementary Figure 5F**). We next investigated second-generation CAR T cells with 4-1BB-based co-stimulation. Again, we observed that the killing capacity of conventional second-generation CARs is slightly decreased when the target cells express CEACAM1^+^ (**Supplementary Figure 6A**). When combining TIM-3-CD28 fusion proteins with conventional second-generation CAR T cells, we saw comparable trends to the combination with first-generation CAR T cells. While the fusion proteins were not able to increase short-term (48 h) killing capacity of second-generation CARs (**Supplementary Figure 6B**), fusion receptor CAR T cells showed higher proliferative potential in the absence of target cells. After the addition of targets, the percent proliferating cells were potentially maxed out at around 80% (**Figure 4E**). The trend of increased T-cell numbers without the addition of targets was again seen in the second-generation CARs (although not significant) when looking at the growth curves (**Supplementary Figure 6C**) and confirmed by analyzing the CAR T-cell counts during the proliferation assay (**Figure 4F**). Surprisingly, the percentage of cytokine-secreting T cells was decreased in fusion receptor second-generation CAR T cells (**Supplementary Figure 6D**). Consistent with the findings in first-generation CARs, we again observed increased levels of CD25 expression (**Figure 4G**) and decreased levels of late-effector phenotype (**Supplementary Figure 6E**) in fusion protein CAR T cells. In summary, TIM-3-CD28 fusion receptor CAR T cells can improve conventional CAR T cells in certain situations. Despite decreased percent cytokine secretion in second-generation CAR T cells, short-term killing is not decreased and TIM-3-CD28 fusion proteins can mediate higher CAR numbers, increased proliferative potential, CD25 expression, and earlier differentiation states of CAR T cells.

## Discussion

Despite high initial response rates in B-cell precursor leukemia and lymphoma, anti-CD19 CAR T-cell therapy can lack long-term efficacy due to multiple factors including limited CAR T-cell proliferation and persistence (3, 27). In recent work, we and others showed that TIM-3 expression on T cells can limit antileukemic T-cell responses both in terms of cytokine release and proliferation (19, 28). Here, we found that TIM-3 gets upregulated on conventional anti-CD19 CAR T cells after a single stimulation with target cells potentially to prevent excessive stimulation. On the other hand, we identified substantial upregulation of the TIM-3 ligand CEACAM1 on leukemic cell lines upon simulation of a Th1 attack. In publicly available RNA-seq data, a correlation in immune cancer between TIM-3 and CEACAM1 expression is seen, which is consistent with reports by other groups that have shown an overexpression of both TIM-3 and CEACAM1 on tumor-infiltrating T cells in a variety of different tumors (29). While the exact impact of CEACAM1 expression and the expression of the other TIM-3 ligands in childhood leukemia is unknown, our group has recently shown that the three protein-based ligands are detectable on RNA level and identified high TIM-3 expression on bone marrow T cells as a prediction marker of dismal prognosis hinting to an important role of the inhibitory TIM-3 axis in ALL (19). We thus decided to generate TIM-3-CD28 fusion receptors to turn TIM-3-mediated inhibition into CD28-based stimulation. For fusion receptor TIM-3-CD28-1, we decided to test a CD8 transmembrane domain as this domain has been used in CAR T cells and shows good surface expression. However, in our experiments, transduction with TIM-3-CD28-1 yielded the lowest transduction rates and geometric mean fluorescent intensity. This is in line with a report by Schlenker et al. (30) who tested PD-1-CD28 fusion proteins and showed that their CD8 transmembrane design led to lowest percent PD-1^+^ cells. The other five TIM-3-CD28 fusion proteins tested here comprised of different portions of TIM-3 and CD28 proteins. As recent reports of CD200R-CD28 fusion receptors have indicated that using larger parts of CD28 including the membrane-proximal extracellular cysteine in amino acid position 141 is superior to other designs (23), we tested TIM-3-CD28 receptors with large CD28 fragments and very short TIM-3 parts, too. To ensure a physiologic distance in the immune synapse between the artificial TIM-3 and its binding partners, we kept the total number of extracellular amino acids stable. In analogy to previous reports of PD-1-CD28 fusion proteins (21), we next tested the activation and proliferation potential of the different fusion receptors by stimulating the T cells with CD3 antibody. While TIM-3-CD28-1 and -2 did not show increased proliferative potential, the two receptors with the largest CD28 parts exerted the highest fold change in proliferation when the percent proliferating cells before/after target cell addition were compared. While the background proliferation (without CD3 stimulation) was <20% for all constructs, it was the lowest for TIM-3-CD28-5 and -6, which contributed to the increased fold change. However, we chose fold change as a readout because the aim was to identify the fusion protein with the highest dynamic range (low background proliferation, strong response to CD3 stimulation). As expected, the proliferative effect was potentiated by adding the soluble form of the different protein-based TIM-3 ligands to the culture. The impact of CEACAM1 addition was rather minimal compared to the other ligands. There are two potential explanations for this finding: 1) We added the soluble version of CEACAM1, and the impact of soluble CEACAM1 on TIM-3 signaling in T cells is not well understood yet. 2) The activated T cells themselves most likely expressed CEACAM1 on the surface, which would dilute the effect of adding additional CEACAM1 to the culture. Increases in proliferative capacity and FC of cytokine release compared to untransduced T cells were observed even without the addition of the ligands. This further underlined the possibility that the activated or bystander T cells could upregulate or secrete the respective ligands. As TIM-3-CD28-5 and -6 also showed the highest dynamic range in cytokine release, we decided to follow up on these two receptors and cloned them into multicistronic constructs in combination with first- and second-generation anti-CD19 CARs. We decided to pair the CD28-based fusion proteins with 4-1BB-mediated costimulation for the second-generation CAR to investigate potential synergistic effects of CD28 and 4-1BB. While the addition of the fusion proteins to first-generation CARs showed slightly increased cytotoxicity against CD19^+^/CEACAM1^+^ target cells, the fusion receptors were not able to increase killing beyond the level of second-generation CARs. Notably, short-term killing assays (48 h) represent prompt effector function, while the expected advantage of the TIM-3-CD28 fusion receptor is pointing toward longevity of T cells. Further *in vivo* studies to evaluate the long-term proliferative and killing capacity would be helpful to analyze the full therapeutic potential of the switch receptors. The strength of the TIM-3-CD28 fusion receptors described here appears to be mostly in terms of proliferation and increased CAR T-cell numbers. Interestingly, without restimulation of the cells, we observed a stronger proliferative advantage of the fusion receptors when paired with a CAR (**Figure 4C**; **Supplementary Figure 6C**) compared to fusion receptors that were not combined with a CAR (**Supplementary Figure 1F**). Reasons for that are speculative but could include differences in soluble or membrane-bound ligand levels, a small amount of tonic signaling through the CAR, or remaining small numbers of CD19^+^ cells in the culture that could provide some background activation to the CAR T cells. While the fusion receptors led to higher CAR numbers and increased proliferation even in the absence of target cells, we did not detect substantial cytokine release without target cell presence. However, the addition of these receptors into CAR T cells might require additional safety considerations, such as suicide switches or synthetic circuits. Further studies are needed to understand the proliferative behavior of TIM-3-CD28 fusion receptors. To clearly dissect the role of the different domains in activating the fusion receptor, mutations could be introduced into the CD28 signaling or the TIM-3/ligand binding domain to disrupt the signal and investigate the specificity and signal transduction of the receptor. Moreover, transcriptional/gene set enrichment analyses will describe the proliferative phenotype and ensure that the increase in proliferation does not lead to long-term dysfunction/exhaustion in the fusion receptor CAR T cells. Overexpressing TIM-3-CD28 fusion proteins together with second-generation CAR T cells led to decreased percentages of IL-2 releasing cells. However, this effect might be outweighed by the increased overall number of CAR T cells with a fusion receptor. The slightly increased percentage of fusion protein positive cells expressing CD62L as a marker of early T-cell differentiation states might create an additional benefit. An interesting finding was the increased levels of CD25 that might render fusion protein CAR T cells more effective in situations of competition for IL-2 or presence of Tregs. Moreover, increased levels of CD25 might have contributed to the proliferative phenotype of the fusion protein CAR T cells. Although they were cultured with minimal levels of exogenous IL-2, the bystander/surrounding CAR T cells most likely produced IL-2, which might have led to a competitive advantage of the fusion protein CAR T cells. In analogy to reports of PD-1-CD28 fusion proteins that seem to increase functionality of low avidity TCRs rather than high avidity TCRs (30), the TIM-3-CD28 fusion receptors might work best in a more challenging setting of, e.g., lower CAR affinity, tonic signaling, or in a solid tumor microenvironment with a high expression of multiple TIM-3 ligands. Genetically modified CAR T cells in combination with checkpoint fusion receptors are a promising treatment alternative to systemic combinations of checkpoint inhibitors together with CAR T cells. While checkpoint inhibitors can cause severe systemic side effects and usually have to be administered multiple times, the fusion receptors will only be expressed specifically on CAR T cells when both the CAR construct and fusion receptor are introduced into the T cell using a polycistronic construct. The rationale is that CAR T cells with a fusion receptor can persist after a single infusion and will not have side effects beyond the known CAR-related complications as shown in recent CAR T-cell trials with PD-1-CD28 fusion proteins (31). Last year, Zhao et al. (26) published a TIM-3-CD28 fusion protein that uses the transmembrane and intracellular domain of CD28 and the extracellular domain of TIM-3 and is thus similar to TIM-3/CD28-4 from our study. Their design mediated increased persistence and antitumor efficacy when combined with a second-generation anti-CD19 CAR. Differences between the two studies include the CAR design as well as the transduction method and the culturing of the cells. While their protocol uses 50 U/ml IL-2, our culturing protocol uses lower levels of IL-2 (6 U/ml) in combination with IL-7 and IL-15, which might have contributed to differences in the two studies. Because Oda etal. (23) had recently shown that using larger parts of CD28 for fusion proteins can be beneficial due to a potential cysteine bond in the extracellular part of CD28, we decided to analyze different portions of CD28 systematically. Our study underlines the finding that including larger parts of CD28 into fusion protein designs might offer possibilities to expand the proliferative potential even further. Thus, the present systematic characterization of TIM-3-CD28 fusion receptors can lay the groundwork for future investigations of these receptors in CAR settings other than the clinically used second-generation anti-CD19 CARs. Further analysis of TIM-3-CD28 fusion proteins could include combination with other CAR specificities, other target cell lines with different expression levels/secretion of the TIM-3 ligands, blocking experiments, as well as co-culture with primary B-precursor blasts. Subsequent evaluation in suitable *in vivo* models (e.g., conventional xenograft or patient-derived xenografts) may reveal additional potential of TIM-3-CD28 fusion proteins.

## Data Availability Statement

The original contributions presented in the study are included in the article/**Supplementary Material**. Further inquiries can be directed to the corresponding author.

## Ethics Statement

The studies involving human participants were reviewed and approved by Ethikkommission bei der Medizinischen Fakultät der LMU München. The patients/participants provided their written informed consent to participate in this study.

## Author Contributions

The approach of the study was set up by TF, FB and SK. Experimental design was done by FB and DS. Fusion proteins were designed by FB, TF and SK. EO, FB, JM, AA, SW, DS, TW, NH and TK performed experiments. FB and TF wrote the manuscript. The manuscript was reviewed by all co-authors. All authors contributed to the article and approved the submitted version.

## Funding

This work was supported by the Kinderkrebshilfe Ebersberg e.V., Deutsche Forschungsgemeinschaft (DFG, German Research Foundation) –SFB- TRR 338/1 2021 –452881907, Gesellschaft für Kinderkrebsforschung, Bettina Bräu Stiftung, and the Gertrud und Hugo Adler Stiftung. SW was supported by the Else-Kröner-Fresenius Stiftung, and DS was supported by the German Cancer Research Center/German Cancer Consortium (DKTK). SK is supported by the Marie-Sklodowska-Curie Program Training Network for Optimizing Adoptive T cell therapy (Grant 955575), funded by the H2020 Program of the European Union, the Hector foundation, the International Doctoral Program i Target: Immunotargeting of Cancer funded by the Elite Network of Bavaria; Melanoma Research Alliance Grants 409510; the German Cancer Aid; the Ernst-Jung-Stiftung (SK); LMU Munich’s Institutional Strategy LMUexcellent within the framework of the German Excellence Initiative (SK); the Bundesministerium für Bildung und Forschung; by the European Research Council Grant 756017, ARMOR-T (to SK), by the German Research Foundation (DFG), the SFB- TRR 338/1 2021 –452881907, the Fritz-Bender-Foundation, and the José-Carreras Foundation. EO was supported by the Kind-Philipp-Stiftung, AA was supported by the Bettina Bräu Stiftung, and JM was supported by the German Cancer Aid (Deutsche Krebshilfe).

## Conflict of Interest

FB and SK: Patent applications have been filed in the field of immuno-oncology. SK has licensed IP to TCR2 Inc, Boston, USA and Carina Biotech, Adelaide, Australia. SK has received research support from TCR2 Inc, Boston, USA and Arcus Biosciences, San Francisco, USA. SK has received honoraria from TCR2 Inc, BMS, and Novartis.

The remaining authors declare that the research was conducted in the absence of any commercial or financial relationships that could be construed as a potential conflict of interest.

## Publisher’s Note

All claims expressed in this article are solely those of the authors and do not necessarily represent those of their affiliated organizations, or those of the publisher, the editors and the reviewers. Any product that may be evaluated in this article, or claim that may be made by its manufacturer, is not guaranteed or endorsed by the publisher.

## Supplementary Material

Supplementary Figure 1 Expression levels of TIM-3, cellular composition, and quality control. **(A)** Leukemia and lymphoma cell lines were stimulated with 100 ng/ml IFN-γ and 10 ng/ml TNF-α for 48 h, and TIM-3 expression was evaluated by flow cytometry with/without stimulation. Experiment was performed in technical triplicates. **(B)** GeoMean fluorescent intensity of TIM-3 on T cells transduced with the different fusion proteins as determined by flow cytometry. N ≥ 3 individual donors. Cellular composition **(C)** and phenotype **(D)** of the T-cell culture 12 days after transduction were analyzed by flow cytometric staining for CD3, CD4, CD8, CD56, c-myc, CD14, and CD19 **(C)** and CD62L, CD45RO, and CD95 **(D)**. N ≥ 3 individual donors. Viability **(E)** and expansion rate **(F)** of transduced T cells were evaluated by trypan blue stain/cell count throughout the culture period. N ≥ 3 individual donors. Data are representative of at least three independent experiments **(B–F)**. Teff, effector T cells; Tem, effector memory T cells; Tcm, central memory T cells; Tscm, stem cell-like memory T cells; Tn, naïve T cells.

Supplementary Figure 2 Proliferation and cytokine release of fusion protein-transduced T cells. **(A)** Fusion receptor-transduced T cells were cultured without prior anti-CD3 coating. Percent proliferating T cells was evaluated by CTV staining. **(B, C)** Background cytokine release of fusion receptor T cells without prior anti-CD3 stimulation. **(D–G)** Fusion protein-transduced T cells were cultured with/without anti-CD3 and the soluble ligands galectin-9 and HMGB1. Cytokine release was determined by intracellular cytokine stain for IFN-γ **(D, E)** and TNF-α **(F, G)**. Experiments were performed in two individual donors and technical duplicates. Data are representative of two independent experiments. Unpaired t-test was performed to determine significance **(D–G)**. FC, fold change; ns, not significant. Physiologic expression levels of TIM-3 ligands are shown in **Supplementary Figure 3**.

Supplementary Figure 3 Expression levels of TIM-3 ligands. Physiologic expression levels of TIM-3 ligands CEACAM1 **(A)**, Galectin-9 **(B)**, and HMGB1 **(C)** were extracted from publicly available RNA-seq data (DICE dataset, https://dice-database.org/).

Supplementary Figure 4 Viability of first- and second-generation CAR T cells. Viability of first-generation **(A)** and second-generation **(B)** CAR T cells with/without fusion proteins was analyzed by trypan blue staining throughout the culture period. N = 2 individual donors. Data are representative of two independent experiments.

Supplementary Figure 5 Efficacy of first-generation CAR T cells with/without fusion proteins. **(A)** First-generation CAR T cells were co-cultured with CEACAM1^+^ or CEACAM1^–^ target cells for 48 h. Cytotoxicity analysis revealed slightly reduced killing capacity against the CEACAM-expressing cell line. N = 2 donors in technical triplicates. **(B)** First-generation CAR T cells with/without TIM-3-CD28 fusion proteins were co-cultured with CEACAM1- target cells, and cytotoxicity was evaluated 48 h later. N = 2 individual donors in technical duplicates. **(C)** First-generation CAR T cells with/without TIM-3-CD28 fusion proteins were co-cultured with CEACAM1+ target cells, and changes in absolute CAR T-cell count were detected by flow cytometry after 72 h. N = 2 individual donors in technical replicates. One-way ANOVA was performed to determine statistical significance. **(D)** CD25 surface expression was evaluated by flow cytometry on first-generation CAR T cells with/without fusion proteins +/- target cells. N = 2 individual donors in technical duplicates. One-way ANOVA was performed to determine statistical significance. **(E)** CD62L expression on first-generation CAR T cells with/without fusion proteins 12 days after transduction. N = 1 donor in technical duplicates. **(F)** T-cell subpopulation of first-generation CAR T cells with/without fusion proteins was analyzed by flow cytometry staining for CD62L, CD45RO, and CD95 on day 12 after transduction. N = 1 donor in technical duplicates. Data are representative of two independent experiments **(A–D)** and of one experiment **(E, F)**, respectively. Teff, effector T cells; Tem, effector memory T cells; Tcm, central memory T cells; Tscm, stem cell-like memory T cells; Tn, naïve T cells; E:T, effector-to-target ratio.

Supplementary Figure 6 Efficacy of second-generation CAR T cells with/without fusion proteins. **(A)** Second-generation CAR T cells were co-cultured with CEACAM1^+^ or CEACAM1^–^ target cells for 48 h. Cytotoxicity analysis revealed slightly reduced killing capacity against the CECAM1-expressing cell line. N = 2 donors in technical triplicates. Data are representative of two independent experiments. **(B)** Killing capacity of second-generation CAR T cells with/without TIM-3-CD28 fusion proteins was analyzed 48 h after starting the co-culture with CD19^+^/CEACAM1^-^ or CEACAM^+^ target cells. N = 1 individual donor in technical duplicates for the 1:1 E/T ratio in the CEACAM^+^ condition and n = 2 individual donors in technical duplicates for all other conditions. Data are representative of at least one independent experiment. One-way ANOVA was performed to determine statistical significance. **(C)** Fold expansion (relative to cell count on day of transduction) was calculated for second-generation CARs +/- fusion protein throughout the culture process. N = 2 individual donors. **(D)** Intracellular cytokine stain was performed after co-culturing second-generation CAR T cells with/without fusion protein with target cells. **(E)** T cell subpopulation of second-generation CAR T cells with/without fusion proteins was analyzed by flow cytometry staining for CD62L, CD45RO, and CD95 on day 12 after transduction. Data are representative of two independent experiments **(C, D)** and of one experiment **(E)**, respectively. Teff, effector T cells; Tem, effector memory T cells; Tcm, central memory T cells; Tscm, stem cell-like memory T cells; Tn, naïve T cells; E:T, effector-to-target ratio; IFN-γ, interferon gamma; TNF-α, tumor necrosis factor alpha; IL-2, interleukin-2.
