# Supplementary material for: Design and Evaluation of TIM-3-CD28 Checkpoint Fusion Proteins to Improve Anti-CD19 CAR T-Cell Function
Source: Front Immunol. 2022 Apr 6;13:845499. doi: 10.3389/fimmu.2022.845499 (PMC9018974; doi:10.3389/fimmu.2022.845499)

Supplementary figure 1 (related to figure 1)

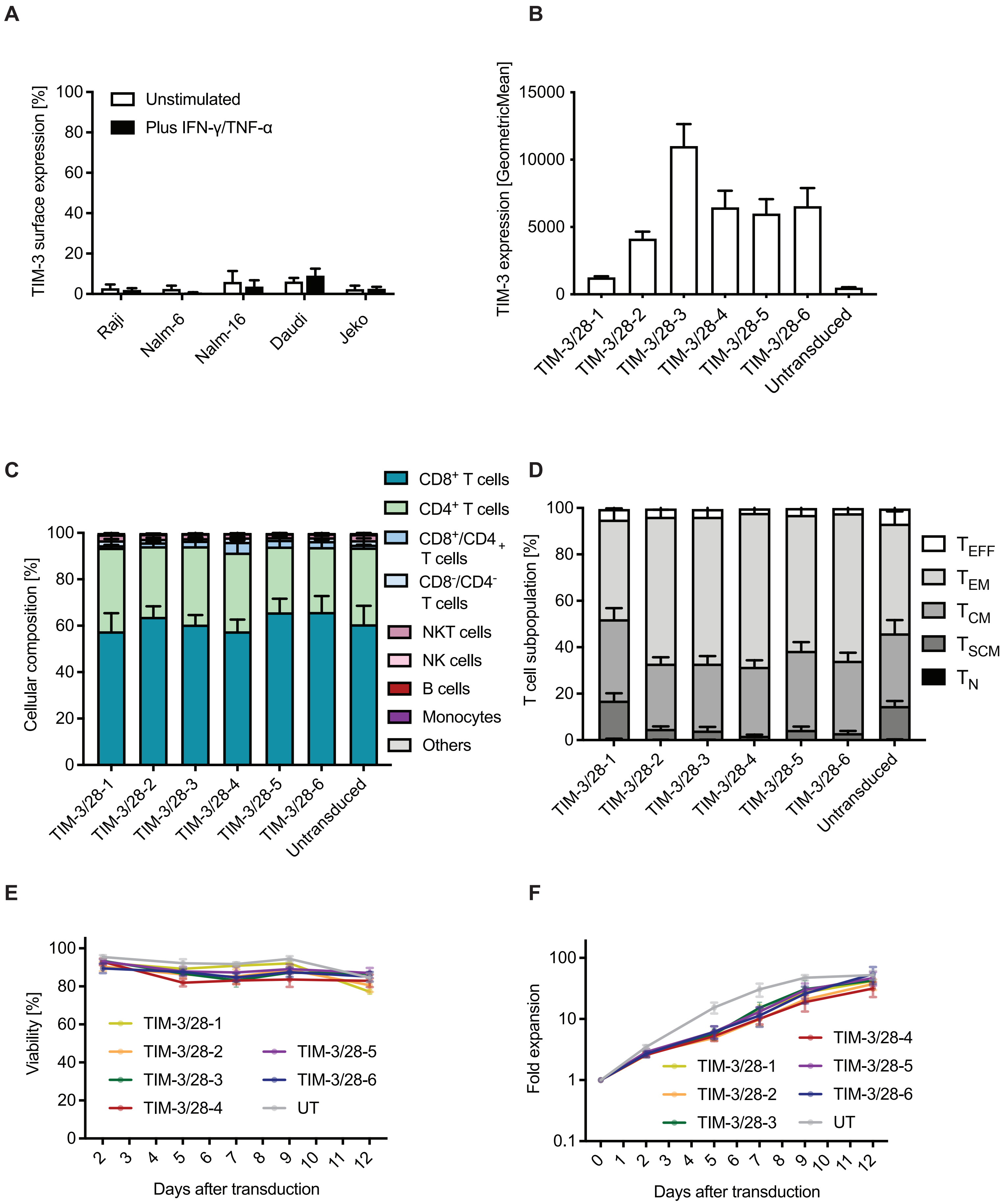

Supplementary figure 2 (related to figure 2)

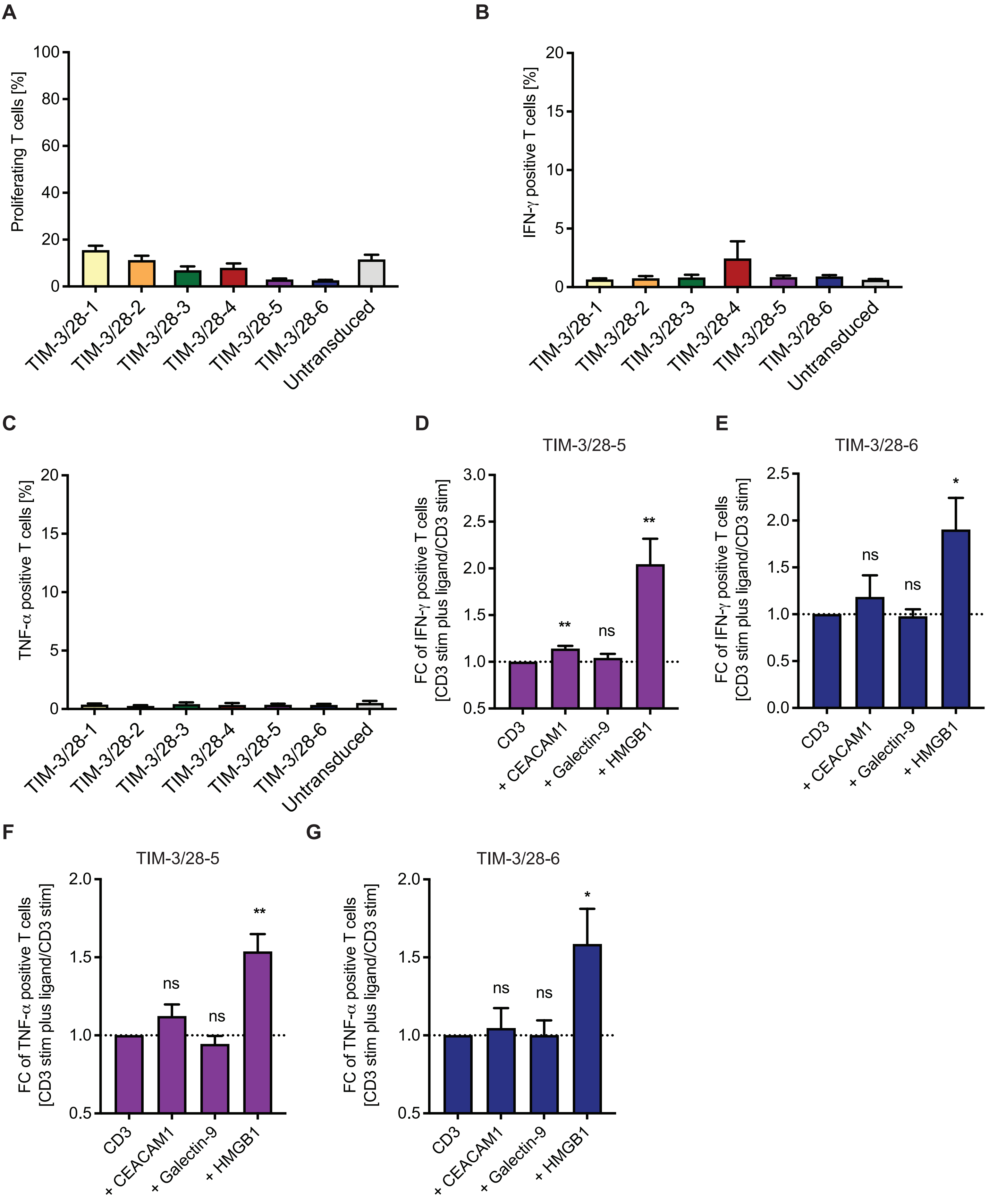

Supplementary figure 3 (related to figure 2)

A

CEACAM1

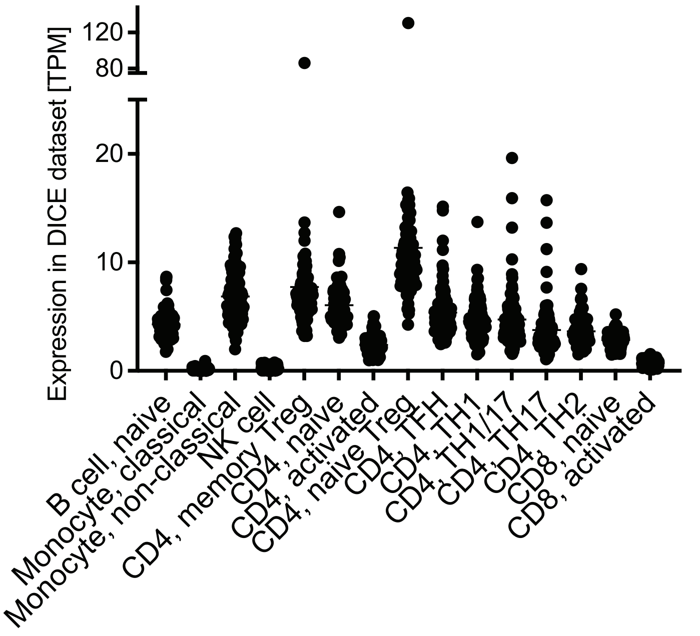

B

Galectin-9

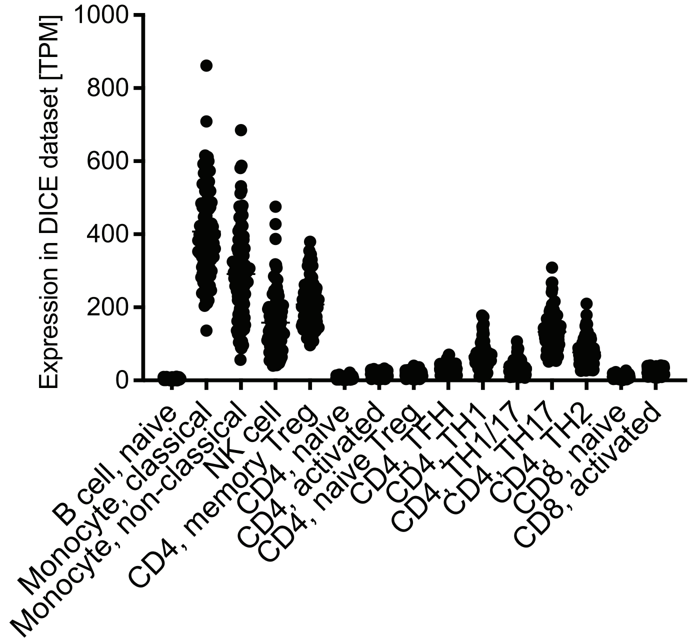

C

HMGB1

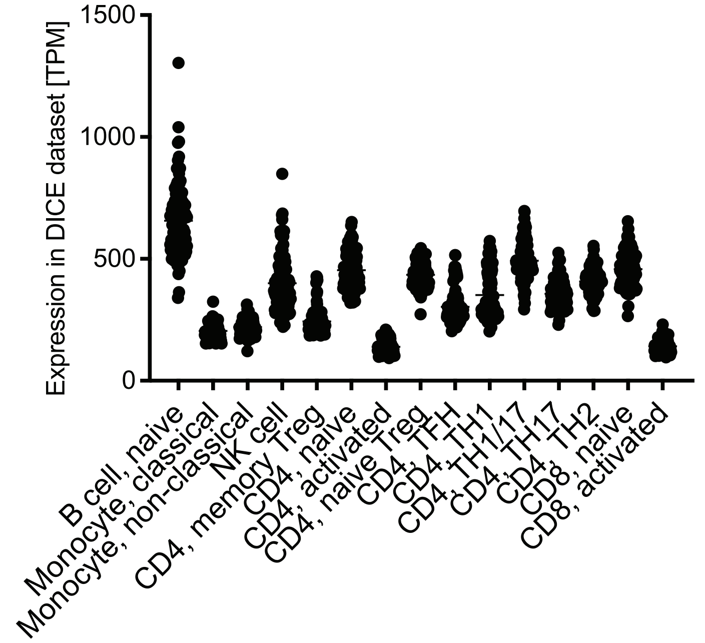

Supplementary figure 4 (related to figure 3)

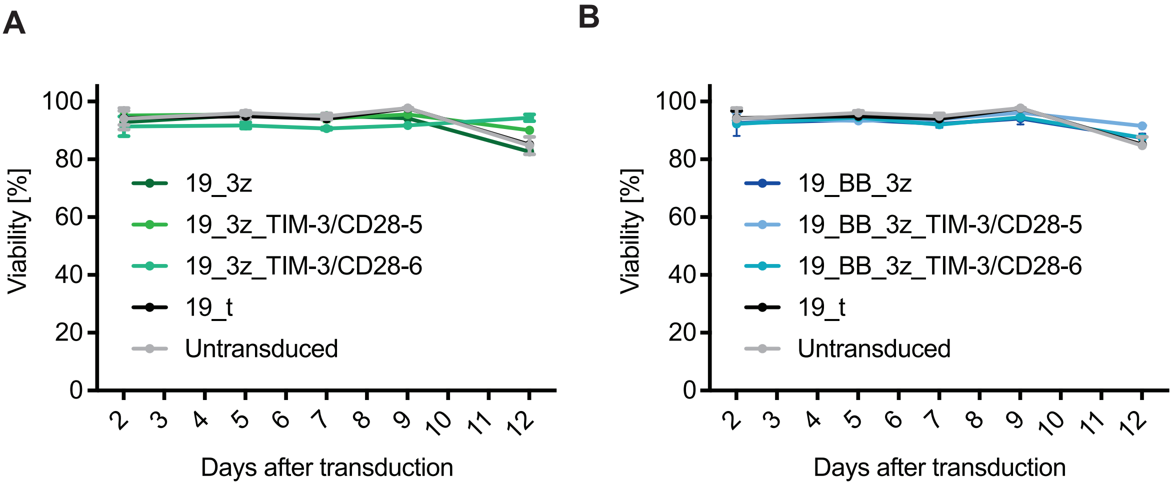

Supplementary figure 5 (related to figure 4)

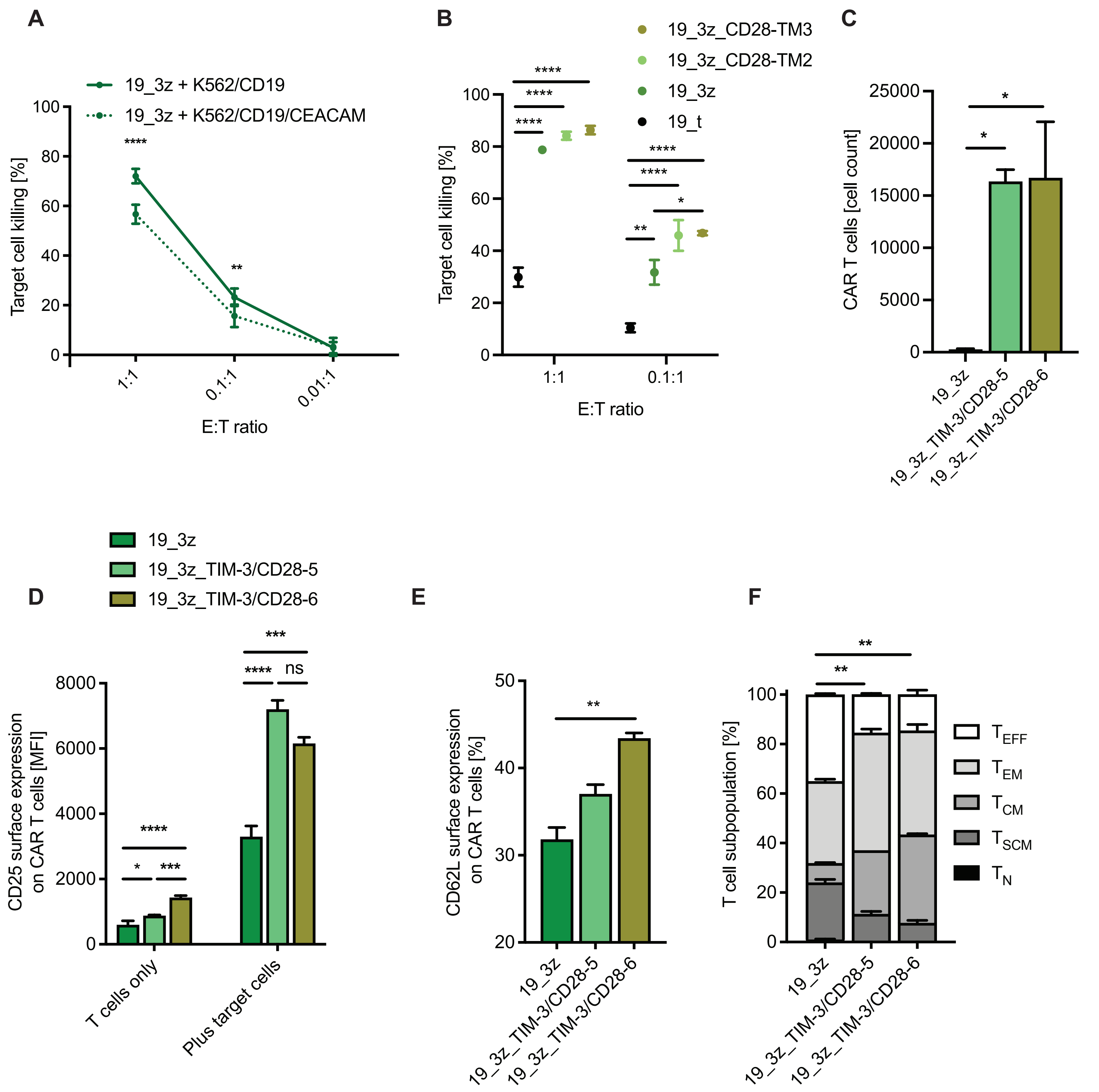

Supplementary figure 6 (related to figure 4)

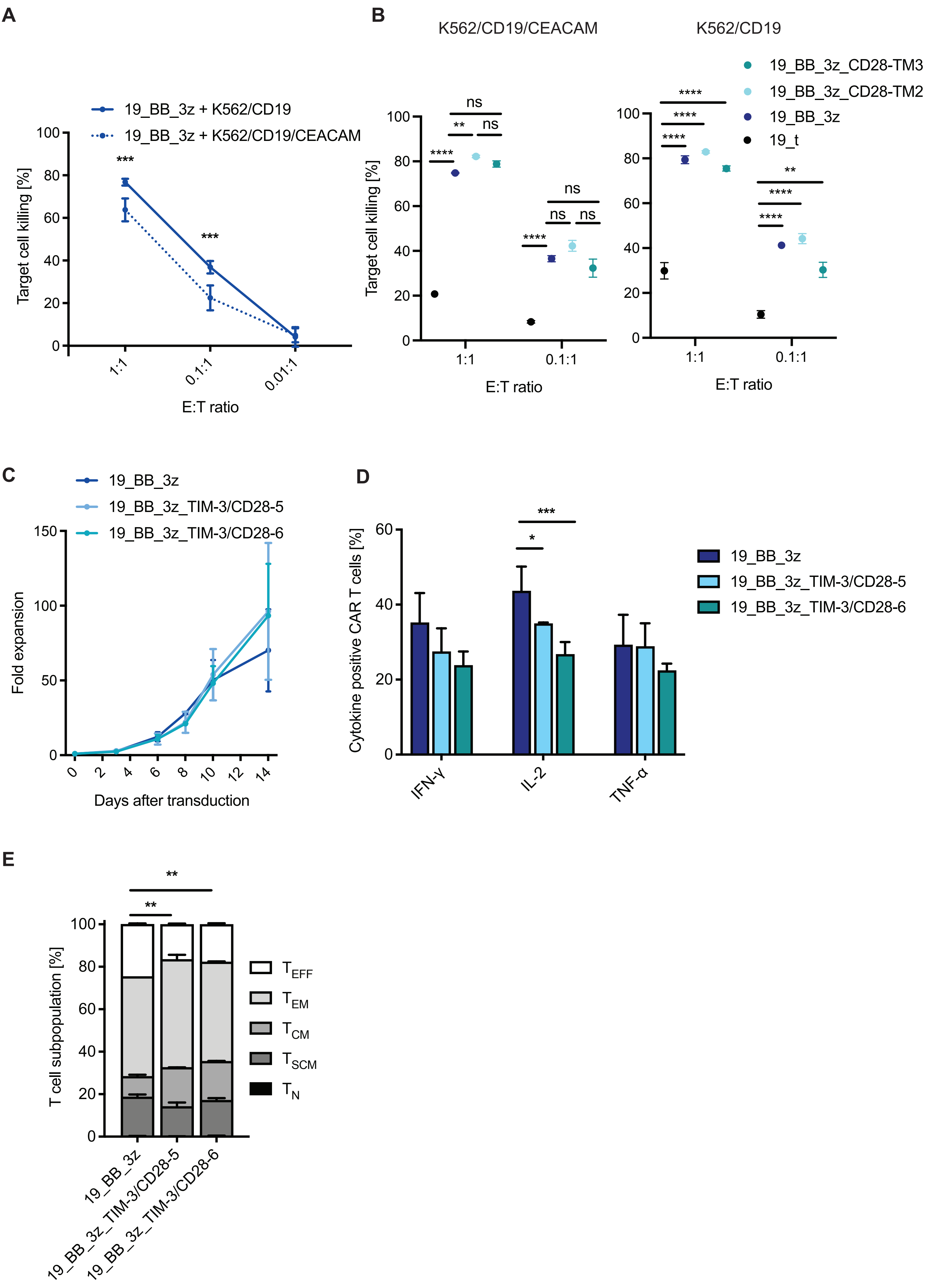

Supplement: Supplementary Figure 1 — Expression levels of TIM-3, cellular composition, and quality control. (A) Leukemia and lymphoma cell lines were stimulated with 100 ng/ml IFN-γ and 10 ng/ml TNF-α for 48 h, and TIM-3 expression was evaluated by flow cytometry with/without stimulation. Experiment was performed in technical triplicates. (B) GeoMean fluorescent intensity of TIM-3 on T cells transduced with the different fusion proteins as determined by flow cytometry. N ≥ 3 individual donors. Cellular composition (C) and phenotype (D) of the T-cell culture 12 days after transduction were analyzed by flow cytometric staining for CD3, CD4, CD8, CD56, c-myc, CD14, and CD19 (C) and CD62L, CD45RO, and CD95 (D). N ≥ 3 individual donors. Viability (E) and expansion rate (F) of transduced T cells were evaluated by trypan blue stain/cell count throughout the culture period. N ≥ 3 individual donors. Data are representative of at least three independent experiments (B–F). Teff, effector T cells; Tem, effector memory T cells; Tcm, central memory T cells; Tscm, stem cell-like memory T cells; Tn, naïve T cells. [file Image_1.pdf]
